# How prepared are students for the various transitions in their medical studies? An Australian university pilot study

**DOI:** 10.15694/mep.2019.000025.1

**Published:** 2019-02-08

**Authors:** Christian Moro, Anne Spooner, Michelle McLean

**Affiliations:** 1 Bond University

**Keywords:** Preparedness for Practice, Transition, Medical Students, Recruitment, Medical Education, Medical Program, Undergraduate Medicine, Postgraduate Medicine, Intrapersonal Skills, Interpersonal Skills, Clinical Skills, Scientific Basis of Disease, Investigations, Student Perceptions, Clinical Preparation, Confidence.

## Abstract

This article was migrated. The article was marked as recommended.

Becoming a doctor involves a series of transitions that require medical students to be equipped with the appropriate knowledge, skills, confidence and professional approach at each step. This pilot cross-sectional study canvassed five cohorts immediately after completing Years 1-5 in a five-year undergraduate medical program (Gold Coast, Australia) regarding their preparedness for the next year. The survey, an amalgamation of two validated inventories, was tailored for each year group to include the expected competencies in five areas. Despite a low response, those who did participate provided valuable information regarding their competence and confidence in terms of their interpersonal and intrapersonal skills, their clinical skills, their ability to apply their theoretical knowledge and investigations. Time management and balancing work and their studies were some areas in which support might be needed. Generally, all student felt comfortable with the communication and physical examination skills but up to half of some of the more senior students were not confident towards dealing with a violent patient and about 20% did not feel at ease communicating and assessing a patient with a mental health issue. Students identified two other areas that requiring curriculum interventions: Working with patients who might be using non-allopathic medications and calculating drug doses. As both impact on patient outcomes, a follow-up study is required. Different recruitment strategies need to be investigated.

## Introduction

Students undergo a series of major transitions on what is often an arduous journey to becoming doctors. Each stage of their individual journey, first as junior or pre-clinical students, then as clinical students in the real world of medicine, requires them to acquire the appropriate knowledge and develop the skills, professional behaviour and attitudes to advance. It behoves us as academics to not only ensure that students are suitably trained for the next stage of their journey, but that they are also sufficiently confident to make the next transition. While there have been a number of studies investigating the transition to the clinical years and the transition to internship following graduation ( Helmich *et al*., 2012; Barbosi *et al*., 2016, Barrett, Trumble and McColl, 2017, Monrouxe *et al*., 2018), much less has been studied in terms of the other transitions on this journey ( Kehoe and Illing, 2017, Moro and McLean, 2017).

Undergraduate medical education is generally characterised by three major transitions for many students: From high school to university ( Barbosi *et al*., 2016), from what is often referred to the ‘pre-clinical phase’ to the ‘clinical phase’ (i.e. working with patients in clinical and community settings) ( Barrett, Trumble and McColl, 2017), and from medical school to the first year of internship ( Wall *et al*., 2006, Monrouxe *et al*., 2018). It is also well documented that medical studies and a medical career are stressful, with a greater proportion of medical students and clinicians suffering burnout and depression compared with the general population ( Rotenstein *et al*., 2016, Boni *et al*., 2018). While the reasons are numerous and include the ‘medical culture’ and exposure to death and dying, medical schools have a responsibility to ensure that students are prepared for the reality of clinical practice ( Kothari, George and Hamid, 2018). Implicit in this assumption is that as educators, we need to know how students perceive their preparedness for each transition so that we can either allay their insecurities and, if necessary, provide remediation to address identified shortcomings and concerns. There is evidence suggesting that those distressed at the beginning of their studies continue to be so throughout their careers ( Nieme and Vainiomaki, 2006). It is therefore imperative to support learners in undergraduate medical programs in particular as they often commence their studies as young school-leavers ( Moro and McLean, 2017). A further risk to consider in the future is the introduction of technology in place of clinical or teacher-directed scenarios, particularly in medical anatomy and physiology ( Moro et al., 2017a, Moro *et al*., 2017b), which is effective at enhancing learning although may result in students becoming even less confident when interacting or communicating information in the clinical environment. Researching how students perceive their preparedness for each transition, some of which are major, such as the transition from medical school to the clinical workplace, would thus be a useful exercise. Despite doing well academically, some learners may falter as they transition from the academic years to the clinical arena which involves multiple abilities across broad competencies that include being part of a multi-professional team, interacting and communicating with patients from diverse cultural backgrounds, dealing with difficult patients, being able to undertake a through physical examination and a range of procedural skills, to understand the rationale for ordering laboratory investigations, diagnose, prescribe and also be able to manage their personal and professional lives ( Prince *et al.,* 2005; Godefrooij, Diemers and Scherpbier, 2010; Atherley *et al.,* 2016).

It should therefore not come as a surprise that anxiety is more common in undergraduate students, i.e. younger medical students ( Hayes *et al*., 2004). Stress and anxiety in the pre-clinical to clinical transition period is common and has been related to a combination of factors related to maturity, professional socialisation ( Helmich *et al*., 2012; Atherley *et al,* 2016), workload, time, and organisation of and deficiencies in knowledge ( Prince *et al.,* 2005; Hayes *et al*., 2004,) Students have described entering the clinical arena as being thrown in the deep end ( Atherley *et al.,* 2016)) as they struggle to apply theoretical knowledge and training in the patient setting and to develop their clinical reasoning skills.

The last major transition for a medical student is from final year medical school to the first year of an internship. Multiple studies support the understanding that many graduates feel unprepared to start work and that preparedness for work varies substantially between medical schools ( Illing *et al*., 2008). Hill and colleagues (1998) in testing eight key areas of adequacy of undergraduate training for internship reported that students from problem-based learning (PBL) curricula were better prepared across most major domains. Wall and colleagues (2006) noted that new doctors felt ill-prepared and ill-equipped for their new role and, identified that the areas of most concern were those of basic doctoring skills such as decision making, prescribing, treatment and practical skills.

### The present study

This study aimed to pilot an online survey designed to canvass all medical students in a five-year programme to identify their perceptions of preparedness for practice for a range of competencies in five domains as they transitioned to the next academic year. The research questions that framed the study were:

• In what competencies and domains did learners perceive that they were well prepared for the next step in their journey?
• In what competencies and domains did learners perceive that they were not well prepared for the next step of their journey?

If competencies and domains were identified for which learners believe they were not prepared, these would be raised at the appropriate year level to investigate further with the view to supporting students better for the transition.

## Methods

### Institutional setting

The pilot study was conducted at Bond University (BU), Gold Coast, Australia and involved Year 1-5 students in the undergraduate medical programme as well as recent graduates (Year 1 interns). Approximately 80% of students are regarded as ‘school-leavers’ as they enter their medical studies directly from high school or have studied for less than 1.5 years at university. The remaining 20% comprises graduate, usually biomedical science graduates or health care professionals such as nurses, dentists, physiotherapists and pharmacists.

The five-year MD programme at Bond University, Gold Coast, Australia, comprises:

• Year 1 (two semesters): Guided, hybrid problem-based learning (PBL)
• Year 2 (three semesters): Hybrid PBL
• Year 3: Case-based learning (CBL), using virtual patients delivered by Bond Virtual Hospital (BVH) and simulation (regarded as a transition year in preparation for clinical practice) ( McLean, Brazil and Johnson 2014)
• Years 4 and 5: Clinical rotations, selectives and electives and the completion of an MD project.

### Study design

The pilot study being reported is part of a larger cross-sectional study designed to prospectively canvass five academic cohorts of learners (Years 1-5) just before writing their final, end of year assessment and then to follow up a few months later once they had transitioned to the next academic year and were settled. They would then be asked retrospectively about their preparedness. For various reasons, not least difficulty recruiting students, the survey remained open from September of one academic year to January the next academic year. Thus, participants had completed their final assessments and were about two weeks into the next academic year. Thus, although this pilot was intended to gauge prospective perceptions of readiness to practice, students were in Years 2-5 and in their first year of internship following graduation.

### Development of the survey instrument

Two validated inventories, the Preparedness for Practice Questionnaires (PHPQ) ( Hill *et al*.,1998) and Illing and colleagues’ (2008) cohort questionnaire were combined and modified to span five domains and which reflected the expected outcomes or competencies at the end of each BU academic year ( Hill *et al*.,1998, Illing *et al*., 2008). Other studies were explored to help define knowledge and skills especially relating to Year 5 and the internship year ( Scicluna *et al.*, 2014, 6. Rolfe *et al.,2002*, Wall *et al*., 2006) as well as the medical school science and scholar, practitioner and health education and professional themes through which curriculum is designed. While the original wording for most items was maintained, some were modified to reflect the Bond University outcomes. The final surveys comprised five domains ( Wall *et al*., 2006, Illing *et al*., 2008), similar to the PHPQ framework ( Hill *et al*.,1998):

• Interpersonal skills (collaboration, communication, teamwork)
• Clinical skills (history and physical examination, patient assessment, procedural skills)
• Investigation, management and preventative care, including prescribing and holistic care (clinical reasoning, patient management)
• Scientific basis of disease (knowledge)
• Intrapersonal skills (confidence, resilience, dealing with uncertainty, time management, professionalism, ethics)

The questionnaire was designed to assess common themes (20 questions) across the entire cohort. Then, additional questions were incorporated that were specific to each year level. Year 1 graduates answered a total of 22 questions; Year 2, 24 questions; Year 3, 37 questions; Year 4, 55 questions; year 5 47 questions; and PGY1, 54 questions. The questions assessed predominantly inter- and intra-personal skills, combined with individualised cohort-specific queries which reflected increasing expertise and competencies across the domains. This allowed each year to be considered individually and in the future would allow tracking of progression of curriculum, and students’ perceptions of their preparedness for transition prospectively and retrospectively. Respondents were asked to rate how confident they were for each statement on a 5-point Likert scale (1 = strongly disagree; 5 = strongly agree.)

### Survey delivery

In the first two weeks of the academic year, in January 2017, all Year 2-5 students and Year 1 interns were sent an email explaining the study and requesting their participation. They were also reminded by the investigators during face-to-face sessions. As the survey was online, a note at the start of the survey informed students and graduates that by completing the survey, it was assumed that they had consented to be part of the study in which their anonymity was maintained. Being at the start of the year, the survey asked students to relate to their perceived preparedness for the attributes that they should have developed in the prior year of study. Informed consent was provided from all participants, and Ethics for the study was approved by the Bond University Human Research Ethics Committee.

## Results/Analysis

### Recruitment and responses

Despite recruiting students in many different ways, e.g. email, personal appeal, etc., the response was poor. Of the 54 students or recently graduated students who initially agreed to participate, 45 responded to a survey with their perceptions towards practice and have been included in this report ( Table 1). Participants responded in five domains (set of skills) regarding their preparation for the next phase of their journeys: Intrapersonal, Interpersonal, Clinical Skills, Scientific basis of disease and Investigations.

**Table 1.** Participant profile across the curriculum and into their internship

Enrolment status		Respondents (%)
*Pre-clinical*	Commencing Year 2	12 (27)
	Commencing Year 3	13 (29)
*Clinical*	Commencing Year 4	4 (9)
	Commencing Year 5	11 (24)
*Internship*	Commencing PGY1	5 (11)
**TOTAL**		**45**

### Perceptions of preparedness

As it is not possible to report all results, only the domains and skills in which learners perceived they were relatively well prepared for practice as well as those they identified as being least prepared will be highlighted.

#### Interpersonal skills

Irrespective of their academic year level, all participants generally believed that they were well prepared in terms of their interpersonal skills such as communicating with patients and working with and respecting team members ( Table 2). An area identified by a considerable number of clinical students that requires attention is dealing with the ‘difficult’ or violent patient. Approximately 20% also identified that they were not prepared for the patient with a mental health issue.

**Table 2.** Students (%) who agreed or strongly agreed with the statement, indicating they were well prepared for the next stage of their studies or professional practice within the interpersonal skills domain

Interpersonal skills	Commencing Year 2 ( *n* = 12)	Commencing Year 3 ( *n* = 13)	Commencing Year 4 ( *n* = 4)	Commencing Year 5 ( *n* = 11)	Commencing PGY1 ( *n* = 5)
I can communicate clearly, sensitively and effectively with patients	100	100	100	100	100
I am able to work as part of a group or team	100	100	100	100	100
I have respect for the roles and expertise of other health and social care professionals	100	100	100	82	100
I am able to work with colleagues with different lifestyles, backgrounds or religions	92	100	100	100	100
I can communicate effectively with colleagues from a variety of health and social care professions	83	100	100	91	100
I am able to deal with difficult and violent patients				73	40
I can communicate with patients who have a mental illness				82	80
I can demonstrate, explain to or teach medical students and colleagues				82	80

#### Intrapersonal skills

While students in all five cohorts generally believed that they were well-prepared in terms of most self-management skills (particularly the PGY1s), some differences across the skills and amongst the cohorts were apparent ( Table 3). For instance, while students were generally confident that they could express their views clearly and could identify their learning needs, at least 20-30% of students across all five cohorts found it difficult to balance their personal lives and their studies and being able to cope with stress. Not surprisingly, time management was also identified as something requiring attention. Noted is the decline in students’ perceptions of their learning in legal and ethical issues.

**Table 3.** Students’ (%) perceptions of their intrapersonal skills

Intrapersonal skills: I am able to:	Commencing Year 2	Commencing Year 3	Commencing Year 4	Commencing Year 5	Commencing PGY1
.. manage my own time effectively	83	92	75	82	100
.. prioritise tasks effectively	92	92	75	91	100
.. perform leadership roles appropriately	75	92	100	91	80
.. identify my own learning needs	92	100	100	91	100
.. cope with stress caused by my work or studies	75	92	75	91	80
.. balance my studies and personal life	67	69	75	82	80
.. remain calm in difficult situations	83	62	100	82	80
..express my views clearly	92	100	100	91	100
I am gaining knowledge of legal and ethical issues (e.g. confidentiality, Mental Health Act)			100	73	80
I manage my health in order to protect patients and colleagues			100	82	80

#### Scientific basis of disease

Table 4 suggests that new Year 3 students felt confident perhaps as they had successfully completed Year 2, a ‘big’ year in terms of content in which all the organ systems are covered, as well as the principles of disease (pathophysiology and pathology). The decline in confidence of Year 4 students may be that once in clinical practice ( *albeit* only a few weeks) they realise how much there is still to know and that it needs to be applied, i.e. being consciously incompetent.

**Table 4.** Students’ (%) perceptions of their ability in terms of integrating and applying their theoretical knowledge

Academic skills	Commencing Year 2	Commencing Year 3	Commencing Year 4	Commencing Year 5	Commencing PGY1
I am able to integrate scientific principles into knowledge of disease	67	92	75	89	100
I am able to apply scientific knowledge to clinical presentations and conditions	NA	100	75	100	100

#### Clinical skills

All cohorts believed that they were well equipped in terms of their history-taking skills ( Table 5). Not surprisingly, the new Year 2 students were not confident in terms of their physical examination skills as Year 1 students would not have practiced these skills. The increasing confidence of the clinical students in terms of physical examination is noted, after 25% of the new Year 4 students (first clinical year) indicated they were not prepared. The variation in the ‘simple practical procedures’ is because what was considered ‘simple’ was changed for each cohort and in fact became more complex. For example, in Year 1, blood pressure was identified, while for Year 4 students, suturing was identified as a ‘simple’ practical procedure. That only half of commencing Year 4 students and 64% of Year 5 students were confident carrying out ‘simple practical procedures is somewhat surprising. For Year 4, it can perhaps be attributed to their ‘newness’ to workplace learning. That only 50% of Year 3 and 73% of Year 4 students did not feel competent performing a mental state examination highlights that some were not confident communicating with a patient with a mental health issue ( Table 2),

**Table 5.** Students’ (%) perceptions of their clinical skills competence

Clinical skills	Commencing Year 2	Commencing Year 3	Commencing Year 4	Commencing Year 5	Commencing PGY1
I can take a patient history	100	100	100	100	100
I can perform a systems-based physical examination	17	100	75	91	100
I can carry out simple practical procedures	100	67	50	64	100
I can perform a full mental-state examination			50	73	100

#### Investigation skills

An area in which where students have expressed an apparent lack of preparedness is in terms of their knowledge of complementary and alternative medicine (CAM) and how this might interfere with allopathic treatment ( Table 6). While there is some engagement in PBL cases, students’ responses suggest that this may be too superficial. About 20-25% of senior and new graduates indicated a difficulty calculating drug doses.

**Table 6.** Students’ (%) perceptions of their ability in terms of a range of investigation skills

Investigation skills I am able to/I can:	Commencing Year 4	Commencing Year 5	Commencing PGY1
.. interpret the results of commonly used investigations	100	100	100
.. assess a patient’s problem	100	100	100
.. formulate a plan to investigate and manage a patient’s problems	75	91	100
.. apply knowledge of alternative and complementary therapies and how these may affect other treatments	50	73	20
.. apply the principles of promoting health and preventing disease	100	91	100
.. apply knowledge of how social and psychological factors impinge on patients’ health and care	100	91	100
.. involve patients in the process of assessing, forming and managing their problems	100	100	100
.. apply safe prescribing for different types of drugs		81	100
.. calculate drug dosages		73	80

*NA = not asked (as not an expected competency)

## Discussion

Despite numerous reminders and requests to students, including personal invitations, the response to this pilot survey was disappointing. We are not alone as curriculum administrators constantly bemoan the fact that fewer than 25% of students complete the end-of-semester evaluation, making it difficult to implement change. Moving forward, we will need to look at different strategies and perhaps incentives to encourage greater participation ( Nair and Merdova, 2008). Involving students as research participants, perhaps as an MD project may be one such strategy.

Not with standing the poor response, this pilot study has provided us with a snapshot of students’ perceptions of their competence (and confidence) for a range of skills in different domains at each transition across a five-year medical program. Overall, from a curriculum perspective, it is satisfying that students believed that for most of the skills, they were prepared for the next stage of their journey, particularly recent graduates. Traditionally, the most emotional transitions are from medical school to the workplace (from Year 3 to Year 4 in the BU Medical Program) and then post-graduation (from Year 5 to interns at BU) ( Helmich et al., 2012). With the BU Year 3 being a transition year, with ‘clinical teams’ undertaking ‘ward rounds’ with virtual patients in the Bond Virtual Hospital and with several opportunities to participate in ward simulations as a member of a multi-professional team in the simulation centre ( McLean, Brazil and Johnson, 2016), students transitioning to the clinical environment in Year 4 would be prepared for a range of tasks and responsibilities with real patients.

This pilot study has identified areas in the BU Medical Program that required attention. Considering the diversity of the Australian population, with 28% being born overseas, with at least 6.8% originating from Asia or South East Asia ( Australian Bureau of Statistics, 2017), any graduating Australian medical student requires a solid foundation of the common CAM and possible interactions with allopathic medications. While BU medical students may have been exposed to CAM theoretically, it was the senior students who were not confident with a patient who may be using alternative therapies. This clearly warrants attention.

With most physicians writing prescriptions for medications, with the need sometimes to adjust doses for age and weight, that about one one-fifth of senior students felt unprepared for this is a red flag that requires addressing as it involves harm to patients. The most likely year would be Year 3, which is the year in which students are applying the theoretical foundations of the previous two years. In the Bond Virtual Hospital, they are suggesting management plans from their ‘patients’ for whom they have discussed a diagnosis or possible diagnoses. The BU medical students are not alone. In South Africa, Harries and Botha (2013) found that only 23% of medical students at the beginning of their third year were competent with these calculations. Sixty-six %, however, achieved competence after an intervention.

Another area in which students identified difficulty was dealing with the violent patient ( Oliver, 2011). With medical school is a safe environment with a high level of support, perhaps it would be appropriate to introduce some complexity and uncertainty earlier in the curriculum to develop a better sense of situational awareness ( Gregory, Hogg and Ker, 2015). A recent commentary by Shapiro (2018) on ‘violence’ in medicine is a useful article on ‘violence’ as a construct and could be used to underpin open discussions with medical students about the different manifestations of violence in the profession. There should certainly be opportunities for students to, through the use of professional actors, engage with such patients and receive appropriate briefings and debriefings. This might start earlier in the curriculum with situational judgement tests, allowing students the liminal space to discuss their reactions.

To a lesser extent, communicating and assessing a patient suffering a mental illness was also reported to be an area where some students are not confident. Similar strategies as suggested above could be used.

## Conclusion

Areas have been identified in which students require additional tuition and support. The most marked in terms of clinical practice include dealing with a violent patient, communicating with a patient who may have a mental illness, calculating drug doses, and being better able to work with patients who may be using CAM. Students may require additional support in terms of the skills related.

Recruitment for medical education research and curriculum evaluation remains an issue, and future studies should aim to enhance participation rates. Strategies need to be investigated to motivate students to participate. As this pilot study has shown, there may be areas of the curriculum that require attention. Better participation is required to ensure that these are indeed issues.

## Take Home Messages

• With medical education is a series of transitions, students need to feel confident in terms of their knowledge and skills at each transition.
• Canvassing students’ preparedness for the various transitions should be undertaken, but ways of engaging students need to be sought.

## Notes On Contributors

**Dr Christian Moro,** BSc, BEd, MBus, PhD, SFHEA

ORCID ID: https://orcid.org/0000-0003-2190-8301

Assistant Professor Christian Moro is the Scientist and Scholar Theme Lead of the Bond University Medical Program. He incorporates a range of technological tools in his lectures and tutorials to enhance student learning. Christian’s research areas include medical education, and laboratory work within biomedical sciences. His current interest revolves around maximising the provision of hands-on activities coupled with online content to assist and enhance university student learning.

**Dr Anne Spooner**, MBBS QLD **.**

Assistant Prof Anne Spooner is a general practitioner and the Practitioner Theme Lead at Bond University. This role encompasses the development, delivery, assessment and evaluation the clinical skills, communication and procedural skills, OSCE and communication programs.

**Professor Michelle McLean**, MSc, PhD, Med, FAMEE

ORCID ID: https://orcid.org/0000-0002-9912-2483

Professor Michelle McLean is currently the Academic Lead for Small Group Learning in the undergraduate medical program at Bond University, Gold Coast, Australia. As a long-standing AMEE member and now an AMEE Fellow, she has been responsible for providing leadership in the Health Professions Education arena, including ensuring avenues of educational scholarship for medical students as part of their studies.
