# Supplementary material for: How prepared are students for the various transitions in their medical studies? An Australian university pilot study
Source: MedEdPublish (2016). 2019 Feb 8;8:25. [Version 1] doi: 10.15694/mep.2019.000025.1 (PMC10712595; doi:10.15694/mep.2019.000025.1)
Supplement: Peer review history

# Reviewer report: Reviewer response for version 1

**DOI:** 10.21956/mep.19769.r29692

**Published:** 2019-02-13

**Authors:** Trevor Gibbs

**Affiliations:** 1 AMEE

This review has been migrated. The reviewer awarded 4 stars out of 5 A very well written paper about an important subject.I find it both interesting and disappointing that we are still researching an area than should, one hopes, be standard practice within all schools and relevant in preparation for any accreditation process. Hence I feel that this is a very useful paper and needs to be read by all those involved at a high level in curriculum design, planning and delivery.Curricula are dynamic entities and must respond to the rapidly changing world of health and social care- it is only through structured evaluation of the outcomes and whether they match the real-world needs that we can ensure a healthier community.

Reviewer Expertise:

NA

No decision status is available

# Reviewer report: Reviewer response for version 1

**DOI:** 10.21956/mep.19769.r29690

**Published:** 2019-02-11

**Authors:** BALAJI ARUMUGAM

**Affiliations:** 1 TAGORE MEDICAL COLLEGE AND HOSPITAL

This review has been migrated. The reviewer awarded 3 stars out of 5 Thanks for inviting to review this article. This article deals with the very important area that is the competencies that the medical graduate should possess at the end of five year programme. This study is unique because it dealt with all the years which facilitated to compare and highlights the increase or decrease of the competencies or the skills among different years. The skills or domains dealt were inter and intra-personal skills and clinical skills. As our medical council of India, suggests the revolutionary approach, which is the competency based curriculum for each subject involving the domains this study was really useful to me as a medical educator and being the curriculum committee member. This study revealed that the clinical skills include dealing with a violent patient, communicating with a patient who may have a mental illness, calculating drug doses were important areas where the study participants were not confident. Suggestions: 1. Being a pilot study, the less sample size is acceptable, but a larger sample would reveal better results. 2. Please try to include Focus group discussions as a part of the large scale study in future to explore more deeper thoughts on the domains or competencies to be learnt by the students.

Reviewer Expertise:

NA

No decision status is available

# Reviewer report: Reviewer response for version 1

**DOI:** 10.21956/mep.19769.r29693

**Published:** 2019-02-11

**Authors:** P Ravi Shankar

**Affiliations:** 1 American International Medical University

This review has been migrated. The reviewer awarded 4 stars out of 5 Thank you for the opportunity to review this interesting paper. The methodology of the study has been well described. The authors describe this as a pilot study. One of the challenges for them would to increase the participation of students. The total number of students has not been mentioned and the percentage of students who participated which is presently unknown can be included. The other issue is whether the authors would like to modify their questionnaire after considering the results of the pilot study. The number of questions was high especially for the later years of study. Complementary and alternative medicines (CAM) and its possible interactions with allopathic treatment could be a challenge considering the diversity of individuals who migrate to Australia. Pharmaceutical calculations can also be a challenge though today software can be of great help. The violent patient can be challenging for students and young doctors. The study is well presented. The article will be of interest to a wide spectrum of medical educators.

Reviewer Expertise:

NA

No decision status is available

# Reviewer report: Reviewer response for version 1

**DOI:** 10.21956/mep.19769.r29691

**Published:** 2019-02-08

**Authors:** Megan Anakin

**Affiliations:** 1 University of Otago

This review has been migrated. The reviewer awarded 3 stars out of 5 Thank you for inviting me to review this article. The authors provide a review of what is known about the transitions undergraduate medical students face during their educational journey into the profession. To address their research question, they chose to use a survey, however, they were disappointed with the limited participation by students at their institution. Despite this limitation, the authors identified several competencies that students reported not feeling well-prepared to demonstrate. In the discussion, the authors relate their results to their programme. To enhance the significance of these results to the reader, the authors might consider relating their results back to the transition issues they identified in the introduction. This study contributes to our understanding about the challenges medical students face as they navigate the transitions in their programme. I look forward to the reading about the next steps and possible interventions the authors put in place to address the issues identified in their study.

Reviewer Expertise:

NA

No decision status is available
